# Add-On Pharmacotherapy in Schizophrenia: Does It Improve Long-Term Outcomes? A Systematic Review

**DOI:** 10.3390/jcm14217847

**Published:** 2025-11-05

**Authors:** Alexandros Smyrnis, Giorgos Smyrnis, Nikolaos Smyrnis

**Affiliations:** 2nd Department of Psychiatry, University General Hospital “ATTIKON”, Medical School, National and Kapodistrian University of Athens, 124 62 Athens, Greece; alsmyrniss150698@gmail.com (A.S.); giorgos.smyrnis@gmail.com (G.S.)

**Keywords:** add-on treatment, schizophrenia, long-term outcome, relapse

## Abstract

**Background/Objectives**: Residual symptoms—such as persistent negative or cognitive symptoms—and relapse remain common in schizophrenia (SCZ) despite the proven efficacy of antipsychotics. As a result, add-on medications are frequently prescribed in real-world clinical practice. Although these agents are often used chronically, most evidence supporting their benefits comes from short-term trials. This systematic review aimed to assess the effect of adjunctive medication on long-term clinical outcomes and relapse prevention. **Methods**: Following PRISMA guidelines, we searched PubMed and Scopus (2000–2025) for trials of add-on agents administered for ≥24 weeks in SCZ spectrum disorders. Eligible studies compared antipsychotic treatment as usual with and without an add-on pharmacological agent (or with an added placebo). The primary outcome was long-term symptom change evaluated via established clinical scales, while relapse was the secondary outcome. Risk of bias was assessed with the Cochrane RoB 2 tool (PROSPERO registration: CRD420251075647). **Results:** The 22 of 4101 selected studies were classified into a group of frequently used add-on agents in clinical practice (antidepressants, mood stabilizers) and a group of less common agents, encompassing cognitive enhancers, antibiotics and antioxidants/anti-inflammatory agents. Results regarding clinical efficacy were mixed for both groups and respective subcategories. Overall, no drug class produced robust benefits. Relapse was systematically reported in only one study, with low overall relapse rates (2.5%). Risk-of-bias assessment did not reveal significant methodological concerns, apart from high attrition (average 29.5%). **Conclusions**: Evidence for the long-term efficacy of add-on pharmacological treatments in SCZ is inconsistent, with no agent demonstrating reliable benefits. These findings raise concerns regarding long-term polypharmacy and also highlight the need for further investigations. Future studies should prioritize longer follow-up, relapse outcomes and realistic treatment patterns.

## 1. Introduction

Schizophrenia (SCZ) is a debilitating illness that affects approximately 1% of the population worldwide. The course of the disorder is chronic, and up to 80% of patients will experience symptom relapse [1], which drastically reduces functioning and overall quality of life. For this reason, current guidelines suggest long-term, or even lifelong treatment with antipsychotics [2]. However, long-term treatment carries the risk of severe side effects, and relapse still occurs in up to 30% of patients despite ongoing antipsychotic use [3]. Furthermore, antipsychotic medication has limited efficacy in controlling certain symptom clusters, such as negative (including blunted affect, avolition, alogia, anhedonia and asociality), cognitive (e.g., deficits in attention or memory) or depressive (e.g., persistent sadness or hopelessness) symptoms. These residual symptoms are highly prevalent and notoriously difficult to manage. Consequently, many clinicians resort to polypharmacy and utilize add-on medications to optimally control symptoms and prevent potential relapses.

The concept of using add-on medication to augment the efficacy of antipsychotics has been investigated for decades. Intuitively, the first agents to be tested were other psychotropic drugs, such as antidepressants [4], benzodiazepines [5] and mood stabilizers [6]. Due to their different mechanisms of action, these agents could act synergistically with antipsychotics ([7,8]). Given the heterogeneous clinical manifestations of SCZ, it has been theorized that these medications could beneficially influence either specific symptom clusters (e.g., antidepressants for depressive symptoms) or overall psychopathology. An often-overlooked distinction in the research of add-on medications is that between benefits for symptom control during an acute episode and prolonged symptom remission during the maintenance phase. Recent reviews of add-on antidepressants in SCZ suggest either mixed results [9] or small improvements in negative and depressive symptoms [10]. Crucially, these conclusions are based on short-term studies; for example, in [9], 33 of 35 trials lasted fewer than 6 months. The same pattern is observed when considering benzodiazepines and mood stabilizers. In a large meta-analysis of antipsychotic augmentation with benzodiazepines [11], it was found that in 16 randomized-controlled trials (RCTs), add-on benzodiazepines had no effect on core symptoms of SCZ. Of the 16 RCTs, none had a duration exceeding 8 weeks, and thus the authors concluded that there is no evidence supporting prolonged benzodiazepine exposure for SCZ patients. A review of add-on mood stabilizers [8] reported conflicting results and highlighted the lack of large, methodologically rigorous studies; notably, only 1 of 34 trials exceeded 6 months in duration.

Apart from established psychotropic agents, investigators have tested a plethora of other compounds, with varying mechanisms of action. Acetylcholinesterase inhibitors such as Rivastigmine, Donepezil and Galantamine, frequently used in dementia, have been utilized to combat cognitive deficits and to improve overall psychopathology, with mixed results [12]. Reviews involving other cognitive enhancers such as Memantine, an NMDA receptor antagonist [13,14], or the wakefulness-promoting agents Modafinil and Armodafinil [15] have not reported high-quality evidence, primarily due to the lack of large, controlled trials. Other agents that have been tested as adjuncts to antipsychotics are amino acids such as N-Acetylcysteine [16], hormonal therapies with Oxytocin [17], anti-inflammatory agents like Aspirin, Celecoxib and omega-3 fatty acids [18,19,20], as well as antibiotics that pass the blood-brain barrier such as D-Cycloserine or Minocycline [21,22] (see [23] for an umbrella review). Although these agents have shown promise, none are currently recommended in treatment guidelines due to insufficient evidence. Notably, in all the above-cited reviews, most findings come from short-term trials, with only 10 of 117 studies equaling or exceeding 6 months in duration.

The pronounced lack of evidence with respect to the long-term efficacy of add-on medications stands in stark contrast to real-world prescribing practices. Large cohort studies have analyzed real-world prescribing data from national registries in the U.S. [24], Asia [25,26,27] and Europe [28,29]. In these studies, the proportion of SCZ patients receiving long-term add-on treatments ranged from about 10% to 80%, depending on country and drug class. This creates the following paradox: patients with SCZ are subjected to polypharmacy, often involving one or more antipsychotic agents coupled with add-on medications. Yet, an overwhelming percentage of evidence for the efficacy of add-on medications originates from short-term trials focused on the acute phase of the disorder. Given the lack of pathophysiological understanding regarding symptom persistence or symptom recurrence after remission, it is conceivable that add-on medications could have beneficial but also potentially detrimental effects on the overall course of chronic disease. At the same time, polypharmacy carries important risks, including increased side-effect burden, pharmacokinetic and pharmacodynamic drug–drug interactions, and reduced adherence due to treatment complexity. These risks are particularly relevant in schizophrenia, where patients are already vulnerable to metabolic, extrapyramidal, anticholinergic and other adverse effects from antipsychotics. These considerations underscore the need to critically evaluate whether add-on strategies truly improve long-term outcomes or merely expose patients to additional harm. The objective of this systematic review was to assess the efficacy of add-on medications regarding long-term clinical outcome and relapse prevention.

## 2. Materials and Methods

This systematic review was conducted in alignment with the PRISMA (Preferred Reporting Items for Systematic Reviews and Meta-Analyses) guidelines ([30,31], see Supplementary Tables S1 and S2, Supplementary Materials pp. 2–6). The following section details the outcomes of the review, the selection procedure and the risk-of-bias assessment of the included studies.

### 2.1. Defining Add-On Treatment

This review concerns trials that use any non-antipsychotic agent to augment treatment as usual with antipsychotics in patients with SCZ spectrum disorders. Treatment as usual refers to all atypical antipsychotics (including long-acting injectables) or haloperidol. All participants were receiving treatment as usual; the comparisons were between treatment as usual plus an add-on agent and treatment as usual plus placebo (or solely treatment as usual).

### 2.2. Outcomes

The primary outcome of the present systematic review is the effect of add-on medication on long-term clinical symptomatology (more than 24 weeks). This threshold was chosen based on the remission criteria proposed by Andreasen et al. [32]. In that consensus paper, symptomatic remission was defined as maintaining sub-threshold symptom scores for a minimum of 6 months. This duration offers a robust benchmark for differentiating short-term symptomatic response from sustained treatment effects and relapse prevention. The primary assessment tool was the Positive and Negative Symptom Scale (PANSS, [33]). PANSS consists of 30 clinician-rated items and provides an overall estimate of disease severity, and is further divided into three subscales appraising positive, negative and general psychopathology. While the most frequently used tool for measuring clinical symptomatology was PANSS, we also considered other scales employed to assess symptoms or functioning. Those were the following: the Hamilton Depression Rating Scale (HAM-D, [34]), the Calgary Depression Scale for Schizophrenia (CDSS, [35]), the Brief Psychiatric Rating Scale (BPRS, [36]), the Scale for the Assessment of Negative Symptoms (SANS, [37]), the Clinical Global Impression scale (CGI, [38]) and the Global Assessment of Functioning (GAF, [39]). HAM-D and CDSS contain 17 and 9 items, respectively, aimed at assessing depressive symptoms. The CDSS is tailored for patients with SCZ. The BPRS is a 15-item tool used to measure a diverse set of symptoms (ranging from emotional withdrawal or hostility to hallucinatory behavior) in patients with psychotic disorders. SANS focuses on negative symptoms in SCZ patients, while CGI is a 3-item questionnaire mainly concerning global disease course. Finally, GAF assesses global functioning, including psychological, social and occupational factors.

Secondarily, we evaluated the potential preventative effect of add-on medication on relapse. We implemented an inclusive definition for relapse, encompassing worsening of symptoms of more than 20% in the applied clinical scale, as well as rehospitalization and suicide. The relative leniency of this definition is due to an observed lack of focus on relapse events in most of the included studies.

### 2.3. Inclusion/Exclusion Criteria

Inclusion and exclusion criteria for the review were designed according to the PICOS/PECOS worksheet [30,31]. To begin with, the present review covers trials using long-term add-on medication as augmentation to antipsychotics. We included studies involving patients with SCZ spectrum disorders, while excluding ones involving other psychiatric disorders such as bipolar disorder or major depressive disorder, as well ones involving patients with SCZ and neurological comorbidities or comorbid alcohol/substance abuse. Furthermore, we excluded studies where add-on medication was administered only to certain patients, on grounds of a specific indication (e.g., benzodiazepines for excessive anxiety). Most studies conducted in a naturalistic setting fall into this category. The minimum duration for a study to be included was set to 24 weeks, since our goal was to focus on long-term outcomes and relapse. We did not include studies implementing non-medicinal adjunctive treatments such as psychotherapy. Lastly, we excluded studies where no clinical assessments were performed, as well as narrative and systematic reviews, meta-analyses, case reports and case series (Table 1).

### 2.4. Selection and Data Extraction Process

The search process started in March 2025 and was concluded in May of the same year. Initially, we compiled a list of add-on compounds that have been used to augment antipsychotics. The list encompasses 70 agents (antidepressants, benzodiazepines, mood stabilizers, cognitive enhancers, anti-inflammatories, etc.; see Supplementary Materials p. 1) and was combined with keywords and phrases such as ‘add-on treatment’ via the use of AND/OR operators to construct a search string which was then applied to the PubMed and Scopus databases. We also applied manual filters for studies in English, published after 1 January 2000. The exact search query is provided in the Supplementary Materials (p. 1). Search results from the two databases were merged into a single Excel spreadsheet and duplicate records were removed. After deduplication, AS and GS independently hand-checked the titles of all remaining records and excluded those that were obviously unrelated to the research objective. At this stage of the selection process, we disregarded papers concerning other psychiatric disorders such as bipolar disorder and major depressive disorder, genetic or molecular psychiatry, neuroimaging and other biomarkers, non-medicinal treatments such as electroconvulsive therapy and animal models. Any discrepancies were resolved by a third independent reviewer (NS). We then compiled a list of relevant titles and retrieved corresponding abstracts and full papers. Those were then evaluated by AS and GS independently, based on our predefined inclusion and exclusion criteria tailored to the PICOS/PECOS worksheet, and the reasons for every exclusion were recorded. Any retrieved reviews that were excluded based on PICOS/PECOS were hand-searched by AS for new, eligible studies, but no unique records fulfilling all inclusion criteria were obtained.

The final selection of studies was then thoroughly analyzed, and the following information was extracted and tabulated: authors, year of publication and country of origin; add-on drug; total daily dosage administered; study duration and design; initial sample and completers; diagnosis of study population; main studied outcomes; core statistical results; and a synthesized view of main findings. Data was extracted by AS and was independently verified by GS and NS. This systematic review was registered beforehand with PROSPERO (CRD420251075647). Excel files and data are available upon request.

### 2.5. Risk-of-Bias Assessment of Included Studies

Risk-of-bias assessments were performed with the use of the revised Cochrane risk-of-bias tool for randomized trials (RoB 2, [40]), by AS and GS independently, with differences resolved by NS. This tool is used to assess one main outcome in each individual study and consists of five domains pertaining to bias in the process of randomization, bias due to deviations from intended interventions, bias due to study attrition, bias regarding outcome measurement and bias due to selective reporting. For each domain, there exists a set of so-called ‘signaling questions’ which are designed to extract core information on various aspects of study design and analysis (see Supplementary Materials, pp. 8–19). Based on the answers to these questions, an algorithm then generates judgements regarding bias arising in that specific domain (Low risk, Some concerns, High risk). The tool also produces an overall judgement, which is ‘High risk’ if one or more domains are deemed high risk and ‘Some concerns’ if one or more domains exhibit ‘Some concerns’ but none are ‘High risk’. Finally, if and only if all domains are ‘Low risk’, then the overall rating is also ‘Low risk’.

## 3. Results

**Figure 1:**
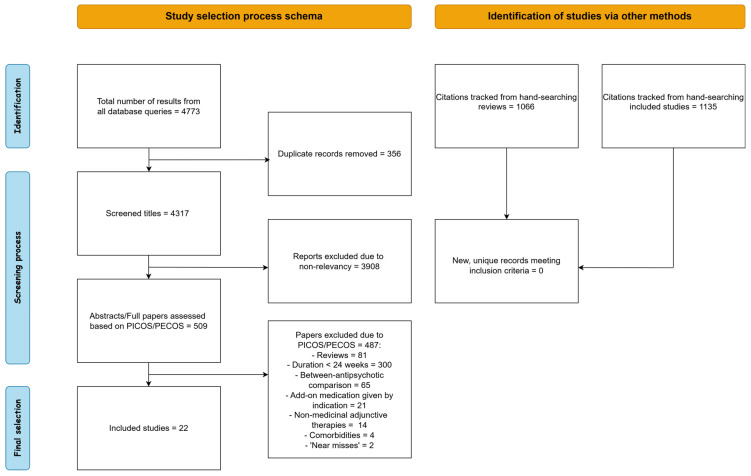
Schematic representation of the selection procedure.

A visual representation of the selection procedure is given in Figure 1. Our search queries in the PubMed and Scopus databases yielded 2250 and 1851 records, respectively, for a total of 4101 records. After deduplication, we scanned the titles of 3745 unique records and excluded 3236 of them due to non-relevancy to the research objective. At this stage of the process, we mainly excluded studies concerning other psychiatric disorders, genetic or molecular psychiatry, neuroimaging, non-medicinal treatments such as ECT and pharmacological mechanisms or interactions, all of which were obviously unrelated to the scope of the present systematic review. The remaining 509 records were meticulously checked against a predefined set of inclusion/exclusion criteria designed according to the PICOS/PECOS worksheet. Based on PICOS/PECOS criteria we excluded 485 studies: 81 reviews and meta-analyses, 300 due to treatment duration of less than 24 weeks, 65 for comparing different antipsychotics rather than add-on medications, 21 for add-on treatment for a specific indication, 14 for using non-medicinal adjunctive therapies such as psychotherapy and 4 for studying patients with neurological comorbidities or comorbid alcohol abuse. Two records ([41,42]) were disregarded as ‘near-misses’ according to PRISMA. While Ref. [41] technically fulfilled inclusion criteria, the study involved the agent Bitopertine, which was since discontinued, based on null results from phase 3 trials [43]. In [42], the authors administered the novel agent Evenamide, primarily to evaluate safety. Even though the agent was given as an add-on to conventional antipsychotics, it is intended as an antipsychotic itself, therefore we did not think it should be classified as an add-on drug. Finally, we hand-search citations from all excluded reviews and all included studies, but obtained no new, eligible records.

We identified a total of 22 unique studies fulfilling all inclusion criteria. To facilitate interpretation, we first split included studies into two groups based on prevalence of use in clinical practice, before adopting a more conventional categorization based on drug class. We classified 8 (36%) studies into the ‘frequently utilized’ group, with 7 (32%) of them testing add-on antidepressants and 1 (4%) testing a mood stabilizer. Of the 14 (64%) of studies classified into the ‘less common/exploratory’ agent group, 6 (28%) investigated add-on cognitive enhancers, 4 (18%) investigated add-on antibiotics and 4 (18%) investigated add-on antioxidants/anti-inflammatory agents.

### 3.1. Frequently Utilized Agents in Clinical Practice (Antidepressants, Mood Stabilizers)

In this section we summarize findings from the 7 (32%) studies using add-on antidepressants and from the 1 (4%) study using an add-on mood stabilizer (Table 2). Three studies using Citalopram, one using both Citalopram and Reboxetine, three using Sertraline and one using Lamotrigine were included.

Friedman et al. [44] conducted a 24-week, double-blind RCT (randomized controlled trial) of Citalopram on top of treatment as usual with antipsychotics against placebo on top of antipsychotics on a sample of 19 SCZ patients, primarily aiming to test for cognitive enhancement, while also delivering clinical assessments. They reported no effect of Citalopram on any PANSS subscale or cognitive metric. In [45], Usall et al. compare both add-on Citalopram and Reboxetine individually against placebo, implementing a 24-week, double-blind, RCT design on 90 SCZ patients with active negative symptoms. Clinical outcome measured by PANSS and SANS was not affected by either Citalopram or Reboxetine. Barnes et al. [46] also found no benefit of add-on Citalopram on negative or total psychopathology over 48 weeks in a double-blind RCT involving 62 SCZ patients exhibiting persistent negative symptoms. Goff et al. [47] reported a marginally significant benefit of add-on Citalopram specific to negative symptoms. The trial was a 52-week, double-blind RCT, with 95 first-episode SCZ patients as participants.

Shi et al. [48] and Zhou et al. [49] studied add-on Sertraline and utilized similar approaches, designing 24-week, double-blind RCTs, the difference being that in [48], 115 treatment-resistant SCZ patients were recruited, whereas in [49], 452 SCZ patients were enrolled during their first episode. Both groups reported significant amelioration in depressive symptoms (HAM-D), as well as in total psychopathology (PANSS total). Positive PANSS was unaffected by Sertraline in both studies. In [48], the authors found significant improvements for negative but not for general PANSS, whereas in [49] it is reported that general, but not negative, psychopathology was affected. Finally, Lang et al. [50] conducted a 24-week, open-label trial of the add-on Sertraline on 230 first-episode SCZ patients. All 4 PANSS subscales and HAM-D scores improved significantly in the Sertraline group.

In the sole retrieved study utilizing an add-on mood stabilizer, Zoccali et al. [51] administered add-on Lamotrigine to 60 treatment-resistant SCZ patients in a 24-week, double-blind RCT. The authors reported significant between-group differences in favor of Lamotrigine at 24 weeks in total SANS, BPRS and CDSS scores.

### 3.2. Experimental Agents

#### 3.2.1. Add-On Cognitive Enhancers

In Table 3, we present the synthesized results from 6 (28%) trials where add-on treatment with cognitive enhancers was administered (three Rivastigmine, one Armodafinil, one Galantamine and one Memantine).

Lenzi et al. [52] performed a 52-week, open-label trial administering add-on Rivastigmine to 16 chronically stable SCZ patients (there was no placebo group), with the primary goal of exploring potential benefits on cognition. While cognitive performance significantly improved with time, BPRS psychotic factors were not affected. Sharma et al. [53] designed a 24-week, double-blind RCT to compare add-on Rivastigmine to placebo in a sample of 21 SCZ patients. No significant time-by-treatment effect was reported for any of the four PANSS subscales or any cognitive performance metric. Lindenmayer et al. [54] tested the potential of add-on Galantamine to long-acting injectable Risperidone as a cognitive enhancer, while also examining clinical symptomatology. Thirty-two patients were enrolled in the 24-week, double-blind randomized, placebo-controlled extension. The authors reported a significant group difference with general PANSS being higher in the Galantamine group. Cognitive metrics were not affected by Galantamine. Kane et al. [55] conducted a 24-week, double-blind RCT with a total sample of 285 SCZ patients exhibiting negative symptoms. They administered add-on Armodafinil at three different dosages of 150, 200 and 250 mg and placebo. ANCOVA revealed no significant differences for any of the three treatment groups compared to placebo in the PANSS total or in the positive and negative subscales. Veerman et al. [56] tested the potential of add-on Memantine against placebo in a 26-week, double-blind, cross-over RCT. The authors reported a marginally significant advantage of Memantine for negative symptoms, while positive and total psychopathology were not affected by Memantine treatment. Lastly, in a 52-week double-blind RCT by Kumar et al. [57], the authors studied the effectiveness of add-on Rivastigmine compared to placebo in 55 stable SCZ patients. The authors reported a significant improvement with time for all PANSS subscales for both groups, and a specific significant effect for the Rivastigmine group regarding the negative and total PANSS.

#### 3.2.2. Add-On Antibiotics

The present segment provides an overview of the results of four (18%) studies utilizing add-on antibiotics, specifically D-Cycloserine (1) and Minocycline (3). More detailed information on these studies can be found in Table 4.

Geoff et al. [58] tested the effectiveness of add-on D-Cycloserine, primarily regarding negative symptoms, in a 24-week, double-blind RCT with a sample of 55 SCZ patients. The mean normalized area under the curve for both total SANS and PANSS did not differ significantly between add-on D-Cycloserine and placebo. Levkovitz et al. [59] used add-on Minocycline in a double-blind RCT with a sample of 54 SCZ patients whose PANSS total score was higher than 60. They reported a significantly better performance of Minocycline versus placebo in the SANS and CGI scales, but no difference in the PANSS total or in any PANSS subscale. Chaudhry et al. [60] conducted a multicenter, 52-week, double-blind RCT studying the effect of add-on Minocycline on 144 stable SCZ patients. The authors reported ameliorative effects on all PANSS scales compared to placebo, with the results being more pronounced in the smaller of the two study centers. Lastly, Deakin et al. [61] implemented a similar design, administering add-on Minocycline on 207 first-episode SCZ patients. The authors reported contradictory results to [60], with no significant time-by-treatment effects in any PANSS subscale or in the CDSS scale. No interaction effect between baseline values and change over time was observed.

#### 3.2.3. Add-On Antioxidants/Anti-Inflammatory Agents

Regarding antioxidants and anti-inflammatory agents, we found a total of 4 (18%) studies, 3 regarding N-Acetylcysteine and 1 regarding omega-3 fatty acids (Table 5).

Pawełczyk et al. [63] tested the effectiveness of supplementation with omega-3 fatty acids in a 24-week, double-blind RCT with 71 first-episode SCZ patients. The authors reported an ameliorative effect versus placebo for general and total PANSS. The same was not observed for positive and negative PANSS. Berk et al. [62] conducted a 24-week, double-blind RCT to assess add-on N-Acetylcysteine compared to placebo. Their sample consisted of 155 chronic SCZ patients, with a PANSS total > 55. Group improvements in favor of N-acetylcysteine were significant for negative, general and total PANSS, but not for positive PANSS. Conus et al. [64] also implemented a 24-week, double-blind RCT design with N-Acetylcysteine. In a sample of 63 early stage SCZ spectrum patients, ANOVA treatment-by-time effects were not significant for any PANSS subscale or for the GAF scale. Finally, in a 52-week, double-blind RCT, Neill et al. [65] also compared add-on N-Acetylcysteine to placebo in a sample of 85 treatment-resistant SCZ spectrum patients. The time-by-group effect for PANSS total, positive and negative scores were insignificant. A marginally significant result was reported for the PANSS depression subscale, defined in the five-factor PANSS model [66].

### 3.3. Relapse

Relapse was explicitly considered as an outcome only in 1 of 24 included studies. In 18 studies, relapse events were marked either in flow diagrams detailing study attrition or in adverse events tables. The remaining 3 studies provided no information regarding the occurrence of relapse.

Overall, 44 of 1770 (2.5%) patients experienced relapse. When considering only studies lasting 6 months, it was 25 of 1168 (2.1%). Aggregating data for all add-on groups and all placebo groups yielded 31 of 944 (3.3%) and 13 of 826 (1.5%) relapsed patients, respectively. Per-compound analysis produced the following results: The number of patients who relapsed was 6 of 67 (8.9%) for Citalopram, 0 of 279 (0%) for Sertraline, 0 of 30 for Lamotrigine (0%), 2 of 55 (3.6) for Rivastigmine, 3 of 15 (20%) for Galantamine, 0 of 27 (%) for D-Cycloserine, 0 of 74 (0%) for N-Acetylcysteine, 1 of 211 (0.05%) for Minocycline, 9 of 213 (4.2%) for Armodafinil and 4 of 26 (15.3%) for Memantine.

Relapses and percentages for individual studies are given in the Tables. Data was too sparse for any meaningful statistical comparison to be performed.

### 3.4. Risk-of-Bias Assessment

A detailed, per-study presentation of the risk-of-bias assessment can be found in the Supplementary Materials (Supplementary Table S3, pp. 8–19). Briefly, we show the aggregated results for each of the five domains of the RoB-2 tool. Regarding bias arising from the randomization process, we categorized 1 study as high risk, meaning that there was no randomization process at all. Another 5 studies were deemed to be medium risk (‘Some concerns’ label), since randomization was stated explicitly, but no description of the exact procedure was provided. The remaining 16 studies were classified as low risk, indicating proper random sequence generation and concealment.

Regarding bias due to deviations from intended interventions, 1 study was assessed as high risk because both participants and raters were aware of the administered intervention. Six studies were given the ‘Some concerns’ label, mainly due to as-treated analyses, or analyses utilizing data only from patients who completed the trial. All the 15 remaining studies were deemed as low risk, implementing double-blinding and intent-to-treat analyses.

Regarding bias due to missing outcome data, we characterized four studies as high risk, because apart from an attrition rate > 25%, we observed that dropout rates either differed drastically between treatment groups, or no information regarding dropout reasons was provided. Fourteen studies were given the ‘Some Concerns’ label, with attrition rates varying from 10 to 60%. In these studies, dropout rates were similar for all treatment groups, which reduces the probability that missing data affected reported outcomes. Four studies were deemed as low risk, with three reporting dropout rates < 10% and one study detailing arguments that data was missing at random. The average attrition rate across all studies was 29.5%. A comprehensive view of study attrition and reasons for dropouts can be found in the Supplementary Materials (Supplementary Figure S1, p. 7).

Regarding bias due to measurement of the outcome, we categorized two studies as having ‘Some concerns’, mainly because raters were not blinded to the intervention, which could have influenced results. The remaining 20 studies were deemed as low risk.

Regarding bias due to selection of reported results, two studies were classified as high risk and two studies were given the ‘Some concerns’ label because of discrepancies between the pre-specified analysis plan and the actual reported results. The remaining 18 studies were low risk, with the analysis plan being in accordance with the presented findings.

**Figure 2:**
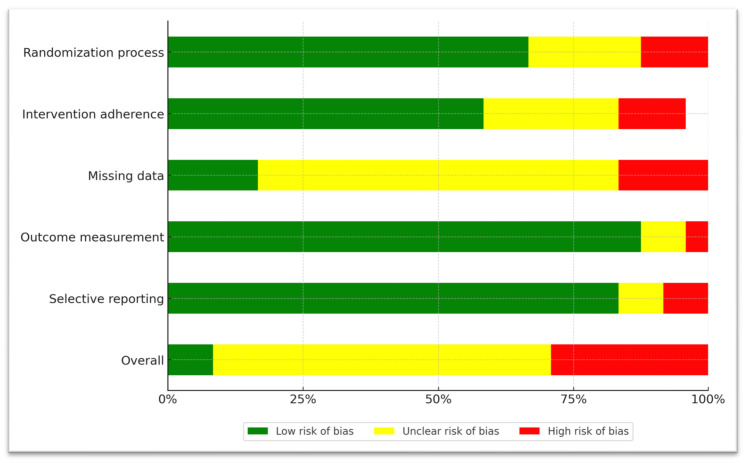
A graphical representation of domain-level, aggregated risk-of-bias data.

These results are summarized in Figure 2 and Table 6.

## 4. Discussion

This systematic review was conducted to evaluate the clinical efficacy of a wide spectrum of add-on pharmacological agents over extended follow-up durations of 6 months or more by synthesizing results from 22 studies. The overarching finding is that, despite decades of investigation and widespread real-world use, the evidence supporting add-on therapies for improving long-term outcomes remains inconclusive and generally weak. While some agents demonstrated modest benefits on specific symptom domains, no compound conferred robust global improvements across studies or reliably reduced relapse rates.

A major concern we would like to emphasize is the pronounced lack of long-term controlled trials involving frequently utilized agents, which comes in stark contrast to real-world prescribing practices. This highlights the urgent need for designing and performing more long-term trials, with a focus on established agents, despite the difficulties that they entail. To contextualize this, we did not find a single study with a long follow-up period on benzodiazepines, while only one such study was retrieved on mood stabilizers. Lamotrigine [51] did offer significant benefits across symptom domains, but the results from a sole study with a relatively small sample size should not be overinterpreted.

In the antidepressant group, we analyzed four studies using add-on Citalopram, and in one of them, there was also a Reboxetine group. The motivation for rigorously testing Citalopram augmentation of antipsychotics originated from theoretical propositions (5-HT dysregulation in SCZ, [67]), symptom overlap with major depressive disorder, as well as empirical findings [68]. Indeed, in one large study [47], Citalopram was found to modestly benefit negative symptoms; however, this finding was not corroborated in other studies [44,45,46], which found no improvement with Citalopram (or with Reboxetine, [45]) in any symptom domain.

Sertraline was the only examined compound which consistently produced ameliorative effects regarding a wide range of symptoms. In two large RCTs [48,49] comparing low-dose Ziprasidone plus Sertraline to normal-dose Ziprasidone, the authors unanimously reported benefits regarding depressive and overall psychopathology. In an open-label trial [50] of Risperidone augmentation with Sertraline, all PANSS subscales were significantly improved. Despite large samples and methodologically sound designs, at least for the first two trials, we note several concerns. Initially, all three studies were conducted exclusively in China, which raises issues of external validity. Moreover, the authors reported no instances of relapse among a total of 797 patients, and the average attrition rate for these three studies was 13%, which is drastically lower than the 29% total average for included studies. This discrepancy is not explained by specific methodological details that would plausibly increase patient retention rate, such as added incentives or one-to-one patient monitoring. Overall, while these positive results merit attention, independent studies across different regions and countries are warranted for further confirmation.

Experimental agents such as cognitive enhancers, antibiotics and anti-inflammatory agents have shown promise in shorter trials, specifically with respect to cognitive and negative symptoms, which often persist despite treatment with antipsychotics. In general, the evidence from the longer follow-up trials we reviewed was mixed, and no individual agent seemed to confer robust benefits.

Cognitive enhancers, in particular, have been utilized in SCZ with the prospect of managing cognitive deficits, which are often non-responsive to treatment as usual. Preliminary results from short-term trials [69] showed cognitive benefits for both Rivastigmine and Galantamine. However, the Rivastigmine trials we reviewed reported conflicting results, with one study demonstrating both clinical and cognitive improvements [57], one study reporting cognitive but not clinical benefits [52] and one study reporting neither [53]. Other cognitive enhancers also produced inconsistent results. In a large trial examining potential cognitive and clinical benefits of the wakefulness-promoting agent Armodafinil [55], null results from a smaller, 6-week study [70] were corroborated, ruling out this drug as an effective adjunct of antipsychotics. Memantine [56], on the other hand, did alleviate negative symptoms, albeit in a small sample.

Add-on antibiotics, particularly Minocycline, generated highly heterogeneous results. The rationale for using Minocycline in psychosis stems from its potential microglia-inhibiting, neuroprotective and NMDA receptor activity [71], as well as empirical evidence predominantly related to negative symptoms [72]. In the present review, findings were conflicting, with one multicenter trial [60] demonstrating broad symptomatic improvements, and another [61] reporting no significant effects. D-Cycloserine [58], an antibiotic and glutamatergic modulator, yielded entirely negative results.

Across four studies involving antioxidants/anti-inflammatory agents, we also observed mixed findings. N-Acetylcysteine, an amino-acid with oxidative stress-reducing properties, presented improvements in all but the positive symptom dimension in [62]. However, results in [64,65] did not corroborate these initial findings. A single study of omega-3 fatty acid supplementation [63] reported benefits across psychopathological domains; however, these findings have not yet been replicated in controlled trials.

Another important finding of this review is the near absence of relapse-focused outcomes. Relapse was systematically assessed in only one trial, and in most others, it could be inferred only indirectly through attrition tables or adverse-event reports. Across all studies, the aggregate relapse rate was low (2.6%), but slightly higher in add-on therapy groups than in placebo groups. Although this difference is not interpretable given the sparse data, it emphasizes that add-on therapy does not appear to provide a clear protective effect against relapse. We identified two possible reasons for the usually low incidence/reporting of relapse events. First, long-term studies generally have high attrition rates (29.5% average). A large percentage of dropouts occurred due to ‘Withdrawn consent’ and ‘Loss to follow-up’ (see Supplementary Figure S1, Supplementary Materials p. 7). It is possible that some of these dropouts were related to worsening of SCZ symptoms. Moreover, many patients refuse to participate in studies in the first place, which could introduce selection bias. (i.e., fewer severely ill patients are likely to participate). To conclude, considering that relapse is a strong determinant of long-term prognosis in schizophrenia, the systematic underreporting of relapse represents a misalignment between clinical need and research practice.

From a clinical perspective, our findings suggest that adjunctive pharmacotherapy should not be routinely prescribed for the purpose of improving long-term outcomes in schizophrenia. A central issue we wanted to draw attention to is the relative lack of evidence regarding the two most important add-on drug classes in SCZ, namely antidepressants and mood stabilizers. We only found one study using a mood stabilizer, while there was no long-term study on various, widely used antidepressants such as Fluoxetine, EsCitalopram and others. Since clinical manifestations such as depressive symptoms or agitation and aggressiveness are very common among SCZ patients, it is of vital importance to ascertain the true effectiveness of these add-on classes in order to formulate detailed guidelines for their usage. In the final analysis, given that polypharmacy is widespread in real-world practice and often involves add-on medications of uncertain benefit, our review provides a cautionary reminder that such strategies should be used judiciously and, ideally, within the context of clinical trials or highly individualized care.

This review has some limitations. First, we restricted our search to PubMed and Scopus and omitted databases such as Embase, Web of Science, and PsycINFO. We judged that the two selected databases provided sufficient coverage of the literature and that expanding the search would yield diminishing benefit relative to the additional screening burden. Second, we underline the lack of evidence regarding many individual agents, or even entire drug classes. Of the 70 agents included in the initial search, we found eligible trials only involving 12. Third, attrition rates were high, even exceeding 40% in some trials. However, we noticed that dropout rates generally did not differ between treatment groups, reducing the probability of bias due to missing data. Additionally, the heterogeneity of patient populations complicates interpretation; some trials recruited first-episode patients, and others treatment-resistant or chronically stable populations, raising concern regarding stage-specific effects. Finally, we observed that in many studies, concomitant benzodiazepine usage was permitted, which could introduce confounding drug–drug interactions. However, given the uniformity of prescribing practices, it is unlikely that the results were affected.

In summary, the available evidence indicates that long-term add-on pharmacological strategies offer limited and inconsistent benefits in schizophrenia. As such, add-on medications should not be considered standard of care but remain an area of active investigation. When add-on treatments are considered as a last resort, for example, in cases of persistent negative or depressive symptoms, they should be introduced cautiously and only as time-limited trials. Clinicians are advised to use the lowest effective dose of the chosen agent and to monitor patients closely for side effects and potential drug–drug interactions. Crucially, treatment response should be reassessed after approximately six months, and if no meaningful clinical improvement is observed, the add-on therapy should be discontinued or modified. Future work on add-on medications should concentrate on adopting longer follow-up periods >12 months, to mirror the chronicity of disease course. Furthermore, real-world practice discrepancies should be addressed, by designing trials that follow polypharmacy patterns observed in registries, while carefully monitoring adherence.

## Figures and Tables

**Table 1:** The Population, Intervention/Exposure, Comparison, Outcome and Study Design (PICOS/PECOS) worksheet.

Parameter	Inclusion	Exclusion
Population	Schizophrenia spectrum disorder patients	Schizophrenia patients with neurological comorbidities or alcohol/substance abuse
Intervention/ Exposure	Add-on, non-antipsychotic medication administered for 24 weeks or more, irrespective of indication	Treatment duration less than 24 weeks, treatment with add-on medication based on specific indications
Comparison	Treatment as usual with atypical antipsychotics or haloperidol	Comparison to non-medicinal adjunctive therapy such as psychotherapy or ECT
Outcome	Clinical scales: PANSS, CDSS, SANS, HAMD, CGI, GAF, BPRS, GAF and relapse %	Solely cognitive assessments
Study Design	Both RCTs and open-label, non-randomized trials	Reviews or meta-analyses, case reports and case series

**Table 2:** Synthesis of studies involving add-on antidepressants and mood stabilizers.

Antidepressants (SSRIs, SNRIs)									
Study (Country)	Add-On Compound	Daily Dosage	Design/Duration	Sample	Diagnosis	Main Outcomes	Statistical Results	Relapse (%)	Synthesis of Main Findings
Friedman et al. 2005 [44] (USA)	Citalopram	20 mg up to 40 mg	24-week, double-blind, randomized, placebo-controlled, cross-over	Total subjects: 19 Completed the 24-week trial: 18 (95%)	SCZ	PANSS, cognitive assessment every 2 weeks till 24 weeks.	ANOVA, for mean difference between baseline and endpoint, placebo and Citalopram PANSS Positive F = 1.15, *p* = 0.29 PANSS Negative F = 0.49, *p* = 0.49 PANSS General F = 1.64, *p* = 0.21	0 (0%)—total	Citalopram had no effect on clinical symptomatology or any cognitive metric in a small sample of SCZ patients.
Usall et al. 2014 [45] (Spain)	Citalopram or Reboxetine	30 mg–8 mg	24-week, double-blind, randomized, placebo-controlled	Total subjects: 90 Completed the 24-week trial: 64 (71%) Citalopram group: Initial: 23 Completed: 17 (73%) Reboxetine group: Initial: 34 Completed: 24 (70%) Placebo group: Initial: 33 Completed: 23 (69%)	SCZ, negative symptoms	PANSS, SANS at baseline, 12 and 24 weeks.	Linear mixed models time by group effect: SANS total F = 0.78, *p* = 0.588 PANSS negative F = 0.83, *p* = 0.553 PANSS positive F = 1.55, *p* = 0.172 PANSS general F = 1.44, *p* = 0.208 PANSS total F = 0.70, *p* = 0.651	No information	Neither Citalopram, nor Reboxetine significantly ameliorated clinical outcome after 6 months of treatment, contrary to short-term past trials.
Barnes et al. 2016 [46] (UK)	Citalopram	20 mg up to 40 mg	48-week, double-blind, randomized, placebo-controlled	Total subjects: 62 Completed the 48-week trial: 36 (58%) Citalopram group: Initial: 30 Completed: 15 (50%) Placebo group: Initial: 32 Completed: 21 (65%)	SCZ, with persistent negative symptoms	PANSS, CDSS at baseline and at 12, 36, 48 weeks	Linear regression showed no significant difference between treatment groups. Between-group differences in: PANSS negative subscale: −1.54 (−4.91 to 1.8, *p* = 0.36) PANSS total: −1.1 (−7.7 to 5.6, *p* > 0.05)	No information	Citalopram did not provide any additional benefit, neither regarding positive and negative symptoms nor in quality of life.
Goff et al. 2019 [47] (USA, China)	Citalopram	20 mg up to 40 mg	52-week, double-blind, randomized, placebo-controlled	Total subjects: 95 Completed the 52-week trial: 51 (53%)	SCZ spectrum, first-episode	CDSS, weekly up to week 8, then every 4 weeks, SANS and BPRS every 4 weeks till 52 weeks.	Linear mixed-model group by visit interaction effect (SE = Standard Error): CDSS difference from baseline −0.7048, SE = 0.3027, t = −2.33, *p* = 0.02 BPRS total, difference from baseline 0.7894, SE = 1.6042, t = 0.49, *p* = 0.62 SANS total, difference from baseline 3.3046, SE = 1.7042, t = 1.94, *p* = 0.05	6 (12.5%)—Citalopram 2 (4.2%)—Placebo	While add-on Citalopram did have a marginally significant effect on negative symptoms, it did not ameliorate global clinical scores.
Shi et al. 2022 [48] (China)	Sertraline	50 mg	24-week, double-blind, randomized, placebo-controlled	Total subjects: 115 Completed the 24-week trial: 100 (86%) Sertraline group: Initial: 53 Completed: 49 (92%) Antipsychotic monotherapy group: Initial: 62 Completed: 51 (82%)	SCZ, treatment resistant	PANSS, HAMD, CGI-S at baseline, 4, 8, 12 and 24 weeks.	Repeated measures ANOVA group by time interaction effects: PANSS negative F = 24.7, *p* < 0.001 PANSS positive F = 0.6, *p* = 0.49 PANSS general F = 2.0, *p* = 0.09 PANSS total F = 4.0, *p* = 0.04 HAM-D F = 16.0, *p* < 0.001	0 (0%)—Sertraline 0 (0%)—Placebo	Add-on Sertraline was found to improve negative and depressive symptoms, as well as total psychopathology. The same was not observed for general and positive psychopathology.
Zhu et al. 2022 [49] (China)	Sertraline	50 mg	24-week, double-blind, randomized, placebo-controlled	Total subjects: 452 Completed the 24-week trial: 406 (89%) Sertraline group: Initial: 226 Completed: 210 (92%) Antipsychotic monotherapy group: Initial: 226 Completed: 196 (86%)	SCZ, first-episode	PANSS, HAMD, CGI-S at baseline, 2, 4, 8, 12 and 24 weeks.	Repeated measures ANOVA group by time interaction effects: PANSS negative F = 1.3, *p* = 0.42 PANSS positive F = 0.9, *p* = 0.38 PANSS general F = 12.3, *p* < 0.001 PANSS total F = 17.9, *p* < 0.001 HAM-D F = 78.3, *p* < 0.001	0 (0%)—Sertraline 0 (0%)—Placebo	Add-on Sertraline produced better results regarding general and depressive symptomatology. The positive and negative dimension improvement in time, did not differ between treatment groups.
Lang et al. 2023 [50] (China)	Sertraline	50 up to 100 mg	24-week, open-label, randomized, placebo-controlled	Total subjects: 230 Completed the 24-week trial: 198 (86%) Sertraline group: Initial: 115 Completed: 109 (94%) Antipsychotic monotherapy group: Initial: 115 Completed: 89 (77%)	SCZ, first-episode	PANSS, HAMD, at baseline, 4, 8, 12 and 24 weeks.	Repeated measures ANOVA group by time interaction effects: PANSS positive F = 8.2, *p* = 0.002 PANSS negative F = 78.0, *p* < 0.001 PANSS general F = 24.2, *p* < 0.001 PANSS total F = 39.2, *p* < 0.001 HAM-D F = 90.5, *p* < 0.001	No information	Sertraline in addition to Risperidone showed significant benefits compared to Risperidone alone, regarding all psychopathological metrics. Placebo effect cannot be ruled out.
Mood Stabilizers									
Zoccali et al. 2007 [51] (Italy)	Lamotrigine	200 mg	24-week, double-blind, randomized, placebo-controlled	Total subjects: 60 Completed the 24-week trial: 51 (85%) Lamotrigine group: Initial: 30 Completed: 26 (86%) Placebo group: Initial: 30 Completed: 25 (83%)	SCZ, treatment resistant	BPRS, CDSS, SANS at baseline, 12 and 24 weeks	Mann–Whitney U test revealed significant between group differences at 24 weeks: SANS total, U = 756, *p* < 0.001, BPRS, U = 109, *p* < 0.001, CDSS, U = 174.5, *p* = 0.004.	0 (0%)—Lamotrigine 0 (0%)—Placebo	Lamotrigine provided long-term clinical benefits, regarding negative and depressive symptoms, as well as overall psychopathology.

**Table 3:** Analysis results for studies using add-on cognitive enhancers such as Acetylcholinesterase inhibitors, NMDA-receptor antagonists and wakefulness promoting agents.

Cognitive Enhancers (Acetylcholinesterase Inhibitors, NMDA Antagonists and Others)									
Study (Country)	Add-On Compound	Daily Dosage	Design/Duration	Sample	Diagnosis	Main Outcomes	Statistical Results	Relapse (%)	Synthesis of Main Findings
Lenzi et al. 2003 [52] (Italy)	Rivastigmine	6 mg	52-week, open-label	Total subjects: 16 Completed the 52-week trial: 10 (62%)	SCZ	BPRS, cognitive assessment at baseline, 4, 8, 12, 16 and 52 weeks.	BPRS psychotic symptoms factors were not affected.	0 (0%)—total	Rivastigmine seemed to improve cognitive symptoms and quality of life, while it did not affect psychotic symptoms. Study results should be interpreted with caution, given the open-label design, and small sample size.
Sharma et al. 2006 [53] (UK)	Rivastigmine	12 mg	24-week, double-blind, randomized, placebo-controlled	Total subjects: 21 Completed the 24-week trial: 21 (100%) Rivastigmine group: Initial: 11 Completed: 11 (100%) Placebo group: Initial: 10 Completed: 10 (100%)	SCZ	PANSS, cognitive assessment at baseline, 12 and 24 weeks	ANOVA revealed neither significant treatment nor time-by-treatment effects for PANSS or any cognitive metric. Time by Treatment: PANSS positive: F = 0.25, *p* = 0.78 PANSS negative: F = 0.98, *p* = 0.40 PANSS general: F = 0.55, *p* = 0.58 PANSS total: F = 0.60, *p* = 0.55	1 (9%)—Rivastigmine 0 (0%)—Placebo	In a small sample, add-on Rivastigmine had no effect compared to placebo in any of the clinical or cognitive variables that were tested.
Lindenmayer et al. 2011 [54] (USA)	Galantamine	24 mg	24-week extension, double-blind, randomized, placebo-controlled	Total subjects: 32 Completed the 24-week trial: 16 (50%) Galantamine group: Initial: 15 Completed: 7 (47%) Placebo group: Initial: 17 Completed: 9 (53%)	SCZ spectrum	5-factor PANSS and cognitive assessment at baseline and at 24 weeks.	ANCOVA model with effects for treatment group, pooled site and baseline value: 2.8-point increase for general psychopathology *p* = 0.002, for Galantamine. Implementing the five-factor model for PANSS, resulted in zero significant between group differences (all *p* > 0.05).	3 (20%)—Galantamine 1 (6%)—Placebo	Cognitive impairments and overall clinical symptomatology did not seem to improve with the addition of Galantamine, albeit in a small sample.
Kane et al. 2012 [55] (USA)	Armodafinil	150, 200 or 250 mg	24-week, double-blind, randomized, placebo-controlled	Total subjects: 285 Completed the 24-week trial: 176 (61%) Armodafinil groups (3): Initial: 213 Completed: 130 (61%) Placebo group: Initial: 72 Completed: 46 (64%)	SCZ, with negative symptoms	PANSS, CGI-S and cognitive assessment at baseline and every 2 weeks till 24 weeks.	Mean (SD) difference between baseline and final visit, for the 3 Armodafinil groups and placebo: 150 mg 200 mg 250 mg Placebo PANSS negative −1.9 (3.75) −2.3 (3.57) −2.0 (3.29) −2.2 (4.08) PANSS positive 0.2 (3.20) 0.2 (3.37) −0.2 (2.78) 0.2 (4.41) PANSS total −2.9 (10.33) −3.5 (10.66) −3.5 (8.51) −3.0 (12.32) All *p* values in ANCOVA were larger than 0.5.	9 (4%)—Armodafinil 3 (4%)—Placebo	Add-on Modafinil exhibited no benefit compared to placebo regarding clinical symptomatology or relapse prevention. This result was the same for all three dosages used in this study.
Veerman et al. 2016 [56] (Netherlands)	Memantine	20 mg	26-week, double-blind, randomized, placebo-controlled, cross-over	Total subjects: 52 Completed the 26-week trial: 47 (87%) Allocated to Memantine group during 1st phase: Initial: 26 Completed: 24 (85%) Allocated to Placebo group during 1st phase: Initial: 26 Completed: 23 (89%)	SCZ, treatment resistant	PANSS, CGI-S, cognitive assessment at 12, 14 and 26 weeks.	Memantine phase in comparison with the placebo phase: PANSS negative symptoms (F = 4.17, ES = 0.29, *p* = 0.043) PANSS positive symptoms (F = 1.008, ES = 0.15, *p* = 0.299) PANSS total symptoms (F = 1.869, ES = 0.19, *p* = 0.174) CGI (F = 0.591, ES = 0.11, *p* = 0.443)	4 (15%)—Memantine 3 (11%)—Placebo	Memantine had a significant effect in reducing negative symptoms specifically, while no difference was reported for positive and total psychopathology.
Kumar et al. 2017 [57] (India)	Rivastigmine	up to 6 mg	52-week, double-blind, randomized, placebo-controlled	Total subjects: 55 Completed the 52-week trial: 48 (87%) Rivastigmine group: Initial: 28 Completed: 24 (85%) Placebo group: Initial: 27 Completed: 24 (89%)	SCZ, stable	PANSS, CGI-I, cognitive assessment at 12, 24, 36 and 52 weeks	Two-way repeated measures ANOVA for PANSS total score. Rivastigmine-Placebo at baseline: 43.1 (12.2)–43.2 (8.1), *p* = 0.99, and at 12 months: 36.1 (6.4)–38.6 (6.2) F = 8.9, *p* < 0.01.	1 (3.5%)—Rivastigmine 0 (0%)—Placebo	Rivastigmine had an added positive impact on positive and negative symptomatology and on some of the assessed cognitive markers.

**Table 4:** Synthesized findings from studies using add-on antibiotics.

Antibiotics									
Study (Country)	Add-on Compound	Daily Dosage	Design/Duration	Sample	Diagnosis	Main Outcomes	Statistical Results	Relapse (%)	Synthesis of Main Findings
Geoff et al. 2004 [58] (USA)	D-Cycloserine	50 mg	24-week, double-blind, randomized, placebo-controlled	Total subjects: 55 Completed the 24-week trial: 26 (47%) D-Cycloserine group: Initial: 27 Completed: 14 (57%) Placebo group: Initial: 28 Completed: 12 (43%)	SCZ, predominant negative symptoms	SANS, PANSS at baseline, week 1, 2, 4 and then every 4 weeks till 24 weeks.	Mean normalized area under the curve for SANS total, from baseline to 24 weeks: D-Cycloserine: 0.93 ± 0.19, Placebo: 0.96 ± 0.12, 95% CI: (−0.12, 0.06) Same results for PANSS.	0 (0%)—D-Cycloserine 0 (0%)—Placebo	D-Cycloserine provided no added benefits compared to antipsychotics alone, on any clinical measure.
Levkovitz et al. 2010 [59] (Israel)	Minocycline	200 mg	24-week, double-blind, randomized, placebo-controlled	Total subjects: 54 Completed the 24-week trial: 21 (39%) Minocycline group: Initial: 36 Completed: 13 (36%) Placebo group: Initial: 18 Completed: 8 (44%)	SCZ, PANSS total > 60	SANS, PANSS, CGI and cognitive assessment at baseline, week 1, 2 and then every 4 weeks till 24 weeks.	Repeated measures ANOVA time-by-treatment interaction effect: SANS total *p* < 0.01, effect size r = 0.46 PANSS negative *p* = 0.43, effect size r = 0.23 PANSS positive *p* = 0.39, effect size r = 0.24 PANSS general *p* = 0.81, effect size r = 0.13 PANSS total *p* = 0.48, effect size r = 0.21 CGI *p* < 0.01, effect size r = 0.61	1 (2.7%)—Minocycline 1 (5.5%)—Placebo	Add-on Minocycline seemed to alleviate negative symptoms measured by the SANS and CGI scales. However, it did not influence the PANSS negative, or any other PANSS symptom dimension.
Chaudhry et al. 2012 [60] (Brazil, Pakistan)	Minocycline	200 mg	52-week, double-blind, randomized, placebo-controlled	Total subjects: 144 Completed the 24-week trial: 94 (65%) Minocycline group: Initial: 71 Completed: 46 (65%) Placebo group: Initial: 73 Completed: 48 (65%)	SCZ spectrum, stable	PANSS, CGI at baseline, 24 and 52 weeks.	Analysis was performed for each center (Pakistan, Brazil). We present the combined ANCOVA treatment effect estimates (95% CI): PANSS negative 3.53 (1.55, 5.51) PANSS positive 2.01 (0.51, 3.50) PANSS general 3.13 (−0.14, 6.41) PANSS total 8.52 (2.57, 14.48)	0 (0%)—Minocycline 2 (2.7%)—Placebo	Negative symptomatology was significantly affected by Minocycline in both study centers. Positive, general, and total symptomatology also improved. However, there was a treatment-by-country effect).
Deakin et al. 2018 [61] (UK)	Minocycline	300 mg	48-week, double-blind, randomized, placebo-controlled	Total subjects: 207 Completed the 48-week trial: 129 (62%) Minocycline group: Initial: 104 Completed: 65 (62%) Placebo group: Initial: 103 Completed: 64 (62%)	SCZ spectrum, first-episode	PANSS, CDSS, GAF at 8, 24, 36, 48 weeks and various cognitive markers and biomarkers.	Random effects regression model time-by-treatment effects (group differences): PANSS negative −0.19 (−1.23 to 0.85), *p* = 0·73 PANSS positive −0.19 (−1.12 to 0.73), *p* = 0·68 PANSS total −0.58 (−3.75 to 2.59), *p* = 0·72 CDSS −0.06 (−0.84 to 0.72), *p* = 0·88	0 (0%)—Minocycline 1 (1%)—Placebo	Minocycline did not influence negative or global symptomatology. This result is unlikely to have been confounded by the relatively low baseline negative symptom scores, given the absence of an interaction effect between baseline values and change over time.

**Table 5:** Overview of studies using Antioxidants and Anti-inflammatory agents.

Antioxidants, Anti-Inflammatory Agents									
Study (Country)	Add-On Compound	Daily Dosage	Design/Duration	Sample	Diagnosis	Main Outcomes	Statistical Results	Relapse (%)	Synthesis of Main Findings
Berk et al. 2008 [62] (Australia, Switzerland)	N-Acetylcysteine	2000 mg	24-week, double-blind, randomized, placebo-controlled	Total subjects: 140 Completed the 24-week trial: 84 (60%) N-Acetylcysteine group: Initial: 69 Completed: 42 (61%) Placebo group: Initial: 71 Completed: 42 (59%)	SCZ, chronic, PANSS total > 55	PANSS, CGI at baseline 2, 4, 6, 8 and then every 4 weeks till 24 weeks.	ANCOVA between treatment group least squares mean difference at 24 weeks: (95% CI) CGI-S 0.32 (0.05, 0.59), *p* < 0.05 PANSS Positive 0.5 (1.1, 2.1), *p* > 0.05 PANSS Negative 1.8 (0.3, 3.3), *p* < 0.05 PANSS General 2.8 (0.2, 5.4), *p* < 0.05 PANSS Total 5.9 (1.5, 10.4), *p* < 0.01	No information	Patients in the N-Acetylcysteine group presented with moderate improvement compared to placebo in all but the positive symptom dimension at the end of the 24-week trial.
Pawełczyk et al. 2016 [63] (Poland)	Omega-3 fatty acids	2200 mg	26-week, double-blind, randomized, placebo-controlled	Total subjects: 71 Completed the 26-week trial: 65 (91%) Omega-3 group: Initial: 36 Completed: 32 (88%) Placebo group: Initial: 35 Completed: 33 (94%)	SCZ, first-episode	PANSS, CDSS, GAF, CGI-S at baseline and at 26 weeks	Mixed-effect models for repeated measures were utilized. Least squares mean differences (i.e., treatment group mean difference—Placebo group mean difference): CDSS: −1.58 (2.7 to 0.47, *p* < 0.01) PANSS positive: −1.09 (2.41 to 0.23, *p* > 0.05) PANSS negative: −0.69 (1.97 to 0.6, *p* > 0.05) PANSS general: −3.14 (5.46 to 0.83, *p* < 0.01) PANSS total: −4.84 (8.77 to 0.92, *p* < 0.05) GAF: −0.32 (−0.64 to −0.01, *p* < 0.05)	No information	The authors reported 6-month improvements in both general psychopathology and global functioning, indicating that omega-3 fatty acids could be a helpful add-on treatment option in maintenance treatment of first-episode patients with antipsychotics.
Conus et al. 2018 [64] (Switzerland, USA)	N-Acetylcysteine	2700 mg	24-week, double-blind, randomized, placebo-controlled	Total subjects: 63 Completed the 24-week trial: 41 (65%) N-Acetylcysteine group: Initial: 32 Completed: 22 (68%) Placebo group: Initial: 31 Completed: 19 (61%)	SCZ spectrum, early-stage, stable	PANSS, GAF, neurocognitive assessment at baseline and every 4 weeks till 24 weeks.	ANOVA treatment-by-time interaction effect for: PANSS negative β = 0.161, SE = 0.237, *p* = 0.50 PANSS positive β = 0.176, SE = 0.207, *p* = 0.39 PANSS general β = 0.598, SE = 0.359, *p* = 0.09 GAF β = −0.332, SE = 0.515, *p* = 0.52	0 (0%)—N-Acetylcysteine 0 (0%)—Placebo	N-acetylcysteine had no effect on any symptomatology or global functioning measure. Previous results may not have been replicated due to the relatively low mean baseline negative symptoms (lack of room for improvement).
Neill et al. 2022 [65] (Australia)	N-Acetylcysteine	2000 mg	52-week, double-blind, randomized, placebo-controlled	Total subjects: 85 Completed the 52-week trial: 43 (51%) N-Acetylcysteine group: Initial: 42 Completed: 21 (50%) Placebo group: Initial: 43 Completed: 22 (51%)	SCZ spectrum, treatment resistant	PANSS, cognitive assessment at baseline, 8, 24 and 52 weeks.	Mixed-model repeated measures, time by group effects: PANSS negative F = 0.6, *p* = 0.616 PANSS positive F = 1.28, *p* = 0.282 PANSS total F = 0.76, *p* = 0.521 PANSS depression F = 2.7, *p* = 0.047	0 (0%)—N-Acetylcysteine 0 (0%)—Placebo	N-acetylcysteine offered no clinical benefit in negative, positive or overall symptomatology. A marginally significant effect was reported for depressive symptoms, which could warrant further investigation.

**Table 6:** Domain-level risk-of-bias assessment of each individual study with the Rob-2 tool. Red circles with minus signs represent the ‘High-risk’ label, yellow circles with question marks represent the ‘Some concerns’ label, and the green circles with the tick mark represent the ‘Low-risk of bias’ label.

Study	Randomization Process	Intervention Adherence	Missing Data	Outcome Measurement	Selective Reporting	Overall Bias
Lenzi et al. 2003 [52]	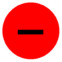	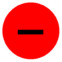	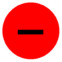	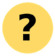	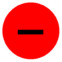	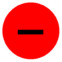
Geoff et al. 2004 [58]	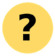	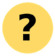	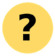	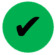	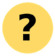	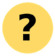
Friedman et al. 2005 [44]	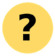	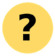	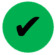	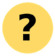	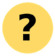	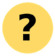
Sharma et al. 2006 [53]	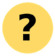	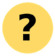	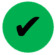	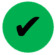	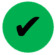	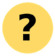
Zoccali et al. 2007 [51]	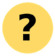	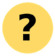	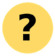	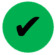	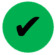	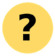
Berk et al. 2008 [62]	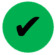	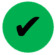	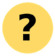	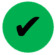	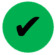	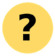
Levkovitz et al. 2010 [59]	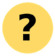	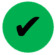	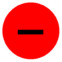	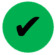	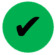	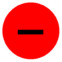
Lindenmayer et al. 2010 [54]	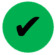	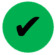	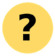	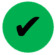	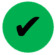	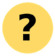
Chaudhry et al. 2012 [60]	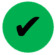	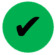	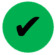	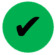	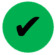	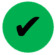
Kane et al. 2012 [55]	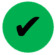	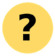	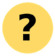	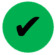	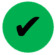	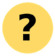
Usall et al. 2014 [45]	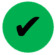	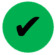	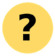	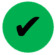	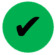	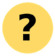
Barnes et al. 2016 [46]	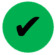	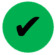	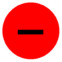	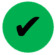	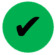	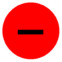
Pawełczyk et al. 2016 [63]	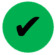	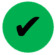	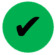	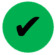	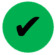	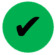
Veerman et al. 2016 [56]	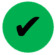	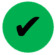	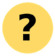	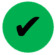	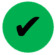	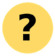
Kumar et al. 2017 [57]	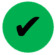	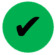	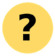	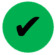	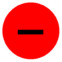	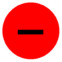
Conus et al. 2018 [64]	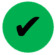	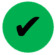	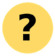	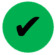	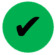	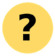
Deakin et al. 2018 [61]	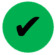	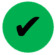	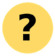	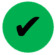	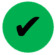	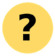
Goff et al. 2019 [47]	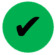	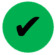	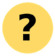	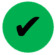	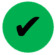	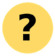
Neill et al. 2022 [65]	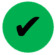	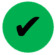	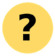	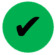	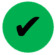	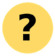
Shi et al. 2022 [48]	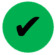	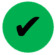	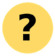	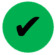	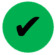	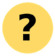
Zhu et al. 2022 [49]	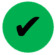	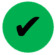	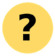	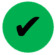	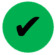	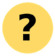
Lang et al. 2023 [50]	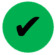	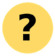	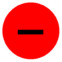	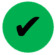	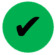	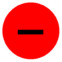

## Data Availability

Data files are available upon request.

## Supplementary Materials

The following supporting information can be downloaded at https://www.mdpi.com/article/10.3390/jcm14217847/s1, Supplementary Table S1: The PRISMA 2020 Checklist; Supplementary Table S2: The PRISMA Abstract Checklist; Supplementary Figure S1: Study attrition breakdown. Reasons for dropout are presented exactly as reported in the original articles; Supplementary Table S3: Detailed risk-of-bias via signaling questions for each individual study (Y = Yes, PY = Probably Yes, NI = No Information, PN = Probably No, N = No, NA = Not Applicable).

## Abbreviations

The following abbreviations are used in this manuscript:

SCZ	Schizophrenia
RCT	Randomized Controlled Trial
PANSS	Positive and Negative Symptom Scale
HAM-D	Hamilton Depression Rating Scale
CDSS	Calgary Depression Scale for Schizophrenia
BPRS	Brief Psychiatric Rating Scale
SANS	Scale for the Assessment of Negative Symptoms
CGI	Clinical Global Impression scale
GAF	Global Assessment of Functioning
