# Supplementary material for: Add-On Pharmacotherapy in Schizophrenia: Does It Improve Long-Term Outcomes? A Systematic Review
Source: J Clin Med. 2025 Nov 5;14(21):7847. doi: 10.3390/jcm14217847 (PMC12608358; doi:10.3390/jcm14217847)
Supplement: Supplementary file 1 [file jcm-14-07847-s001.zip › jcm-3913990-supplementary.pdf]

Exact search query used in the PubMed and Scopus advanced search tool:

((add on therapy) OR (adjunctive therapy) OR (supportive therapy) OR (adjunctive pharmacotherapy) OR (adjunctive treatment) OR (add on pharmacotherapy) OR (add on treatment) OR (supportive treatment) OR (SSRIs) OR (Sodium benzoate) OR (Cannabidiol) OR (Oxytocin) OR (Amantadine) OR (Estradiol) OR (Raloxifene) OR (Anti-inflammatory) OR (Pioglitazone) OR (Celecoxib) OR (Aspirin) OR (Minocycline) OR (Omega-3 fatty acids) OR (Glutamatergic) OR (N-Acetylcysteine) OR (D-Cycloserine) OR (Sarcosine) OR (Glycine) OR (D-Serine) OR (Memantine) OR (Beta-Blockers) OR (Propranolol) OR (Nadolol) OR (Atenolol) OR (Anticholinergics) OR (Trihexyphenidyl) OR (Benztropine) OR (Biperiden) OR (Diphenhydramine) OR (Cognitive Enhancers) OR (Galantamine) OR (Rivastigmine) OR (Donepezil) OR (Armodafinil) OR (Modafinil) OR (Anxiolytics) OR (Benzodiazepines) OR (Lorazepam) OR (Clonazepam) OR (Diazepam) OR (Alprazolam) OR (Oxazepam) OR (Chlordiazepoxide) OR (Temazepam) OR (Zolpidem) OR (Zaleplon) OR (Eszopiclone) OR (Antidepressants) OR (SNRIs) OR (NDRIs) OR (TCAs) OR (MAOIs) OR (Selegiline) OR (Isocarboxazid) OR (Tranylcypromine) OR (Phenelzine) OR (Clomipramine) OR (Desipramine) OR (Imipramine) OR (Nortriptyline) OR (Amitriptyline) OR (Mirtazapine) OR (Bupropion) OR (Milnacipran) OR (Duloxetine) OR (Desvenlafaxine) OR (Venlafaxine) OR (Fluvoxamine) OR (Escitalopram) OR (Citalopram) OR (Paroxetine) OR (Sertraline) OR (Fluoxetine) OR (Mood Stabilizers) OR (Lithium carbonate) OR (Lithium citrate) OR (Divalproex sodium) OR (Valproic acid) OR (Carbamazepine) OR (Oxcarbazepine) OR (Lamotrigine) OR (Topiramate) OR (Gabapentin) OR (Pregabalin)) AND ((schizophrenia) OR (schizophrenia spectrum disorders) OR (psychosis) OR (psychotic spectrum disorders)) AND (relapse)

Supplementary Table S1. The PRISMA 2020 Checklist.

Section and Topic	Item #	Checklist item	Location where item is reported
**TITLE**			
Title	1	Identify the report as a systematic review.	Page 1
**ABSTRACT**			
Abstract	2	See the PRISMA 2020 for Abstracts checklist.	Page 1
**INTRODUCTION**			
Rationale	3	Describe the rationale for the review in the context of existing knowledge.	Pages 2-3
Objectives	4	Provide an explicit statement of the objective(s) or question(s) the review addresses.	Page 3
**METHODS**			
Eligibility criteria	5	Specify the inclusion and exclusion criteria for the review and how studies were grouped for the syntheses.	Page 4
Information sources	6	Specify all databases, registers, websites, organizations, reference lists and other sources searched or consulted to identify studies. Specify the date when each source was last searched or consulted.	Page 5
Search strategy	7	Present the full search strategies for all databases, registers and websites, including any filters and limits used.	Page 5
Selection process	8	Specify the methods used to decide whether a study met the inclusion criteria of the review, including how many reviewers screened each record and each report retrieved, whether they worked independently, and if applicable, details of automation tools used in the process.	Pages 4-5
Data collection process	9	Specify the methods used to collect data from reports, including how many reviewers collected data from each report, whether they worked independently, any processes for obtaining or confirming data from study investigators, and if applicable, details of automation tools used in the process.	Pages 4-5
Data items	10a	List and define all outcomes for which data were sought. Specify whether all results that were compatible with each outcome domain in each study were sought (e.g. for all measures, time points, analyses), and if not, the methods used to decide which results to collect.	Pages 3-4

	10b	List and define all other variables for which data were sought (e.g. participant and intervention characteristics, funding sources). Describe any assumptions made about any missing or unclear information.	Page 5
Study risk of bias assessment	11	Specify the methods used to assess risk of bias in the included studies, including details of the tool(s) used, how many reviewers assessed each study and whether they worked independently, and if applicable, details of automation tools used in the process.	Pages 5-6
Effect measures	12	Specify for each outcome the effect measure(s) (e.g. risk ratio, mean difference) used in the synthesis or presentation of results.	/
Synthesis methods	13a	Describe the processes used to decide which studies were eligible for each synthesis (e.g. tabulating the study intervention characteristics and comparing against the planned groups for each synthesis (item #5)).	Page 4
	13b	Describe any methods required to prepare the data for presentation or synthesis, such as handling of missing summary statistics, or data conversions.	Pages 4-5
	13c	Describe any methods used to tabulate or visually display results of individual studies and syntheses.	Pages 4-5
	13d	Describe any methods used to synthesize results and provide a rationale for the choice(s). If meta-analysis was performed, describe the model(s), method(s) to identify the presence and extent of statistical heterogeneity, and software package(s) used.	/
	13e	Describe any methods used to explore possible causes of heterogeneity among study results (e.g. subgroup analysis, meta-regression).	/
	13f	Describe any sensitivity analyses conducted to assess robustness of the synthesized results.	/
Reporting bias assessment	14	Describe any methods used to assess risk of bias due to missing results in a synthesis (arising from reporting biases).	/
Certainty assessment	15	Describe any methods used to assess certainty (or confidence) in the body of evidence for an outcome.	/
**RESULTS**			
Study selection	16a	Describe the results of the search and selection process, from the number of records identified in the search to the number of studies included in the review, ideally using a flow diagram.	Pages 6-7
	16b	Cite studies that might appear to meet the inclusion criteria, but which were excluded, and explain why they were excluded.	/
Study characteristics	17	Cite each included study and present its characteristics.	Pages 7-18

Risk of bias in studies	18	Present assessments of risk of bias for each included study.	Pages 19-21, and Suppl.
Results of individual studies	19	For all outcomes, present, for each study: (a) summary statistics for each group (where appropriate) and (b) an effect estimate and its precision (e.g. confidence/credible interval), ideally using structured tables or plots.	Pages 7-18
Results of syntheses	20a	For each synthesis, briefly summarise the characteristics and risk of bias among contributing studies.	/
	20b	Present results of all statistical syntheses conducted. If meta-analysis was done, present for each the summary estimate and its precision (e.g. confidence/credible interval) and measures of statistical heterogeneity. If comparing groups, describe the direction of the effect.	/
	20c	Present results of all investigations of possible causes of heterogeneity among study results.	Page 22
	20d	Present results of all sensitivity analyses conducted to assess the robustness of the synthesized results.	/
Reporting biases	21	Present assessments of risk of bias due to missing results (arising from reporting biases) for each synthesis assessed.	/
Certainty of evidence	22	Present assessments of certainty (or confidence) in the body of evidence for each outcome assessed.	Section 22-24
**DISCUSSION**			
Discussion	23a	Provide a general interpretation of the results in the context of other evidence.	Pages 22-24
	23b	Discuss any limitations of the evidence included in the review.	Page 23
	23c	Discuss any limitations of the review processes used.	Page 24
	23d	Discuss implications of the results for practice, policy, and future research.	Pages 23-24
**OTHER INFORMATION**			
Registration and protocol	24a	Provide registration information for the review, including register name and registration number, or state that the review was not registered.	Page 5
	24b	Indicate where the review protocol can be accessed, or state that a protocol was not prepared.	Page 5
	24c	Describe and explain any amendments to information provided at registration or in the protocol.	/
Support	25	Describe sources of financial or non-financial support for the review, and the role of the funders or sponsors in the review.	Page 24

Competing interests	26	Declare any competing interests of review authors.	Page 24
Availability of data, code and other materials	27	Report which of the following are publicly available and where they can be found: template data collection forms; data extracted from included studies; data used for all analyses; analytic code; any other materials used in the review.	Page 24

From: Page MJ, McKenzie JE, Bossuyt PM, Boutron I, Hoffmann TC, Mulrow CD, et al. The PRISMA 2020 statement: an updated guideline for reporting systematic reviews. BMJ 2021;372:n71. doi: 10.1136/bmj.n71

For more information, visit: http://www.prisma-statement.org/

Supplementary Table S2. The PRISMA Abstract Checklist.

Section and Topic	Item #	Checklist item	Reported (Yes/No)
**TITLE**			
Title	1	Identify the report as a systematic review.	YES
**BACKGROUND**			
Objectives	2	Provide an explicit statement of the main objective(s) or question(s) the systematic review addresses.	YES
**METHODS**			
Eligibility criteria	3	Specify the inclusion and exclusion criteria for the systematic review.	YES
Information sources	4	Specify the information sources (e.g. databases, registers) used to identify studies and the date when each was last searched.	YES
Risk of bias	5	Specify the methods used to assess risk of bias in the included studies.	YES
Synthesis of results	6	Specify the methods used to present and synthesise results.	YES
**RESULTS**			
Included studies	7	Give the total number of included studies and participants and summarise relevant characteristics of studies.	YES
Synthesis of results	8	Present results for main outcomes, preferably indicating the number of included studies and participants for each. If systematic review was done, report the summary estimate and confidence/credible interval. If comparing groups, indicate the direction of the effect (i.e. which group is favoured).	YES
**DISCUSSION**			
Limitations of evidence	9	Provide a brief summary of the limitations of the evidence included in the systematic review (e.g. study risk of bias, inconsistency and imprecision).	YES
Interpretation	10	Provide a general interpretation of the results and important implications.	YES
**OTHER**			
Funding	11	Specify the primary source of funding for the systematic review.	NO
Registration	12	Provide the register name and registration number.	YES

Supplementary Figure S1. Study attrition breakdown. Reasons for dropout are presented exactly as reported in the original articles.

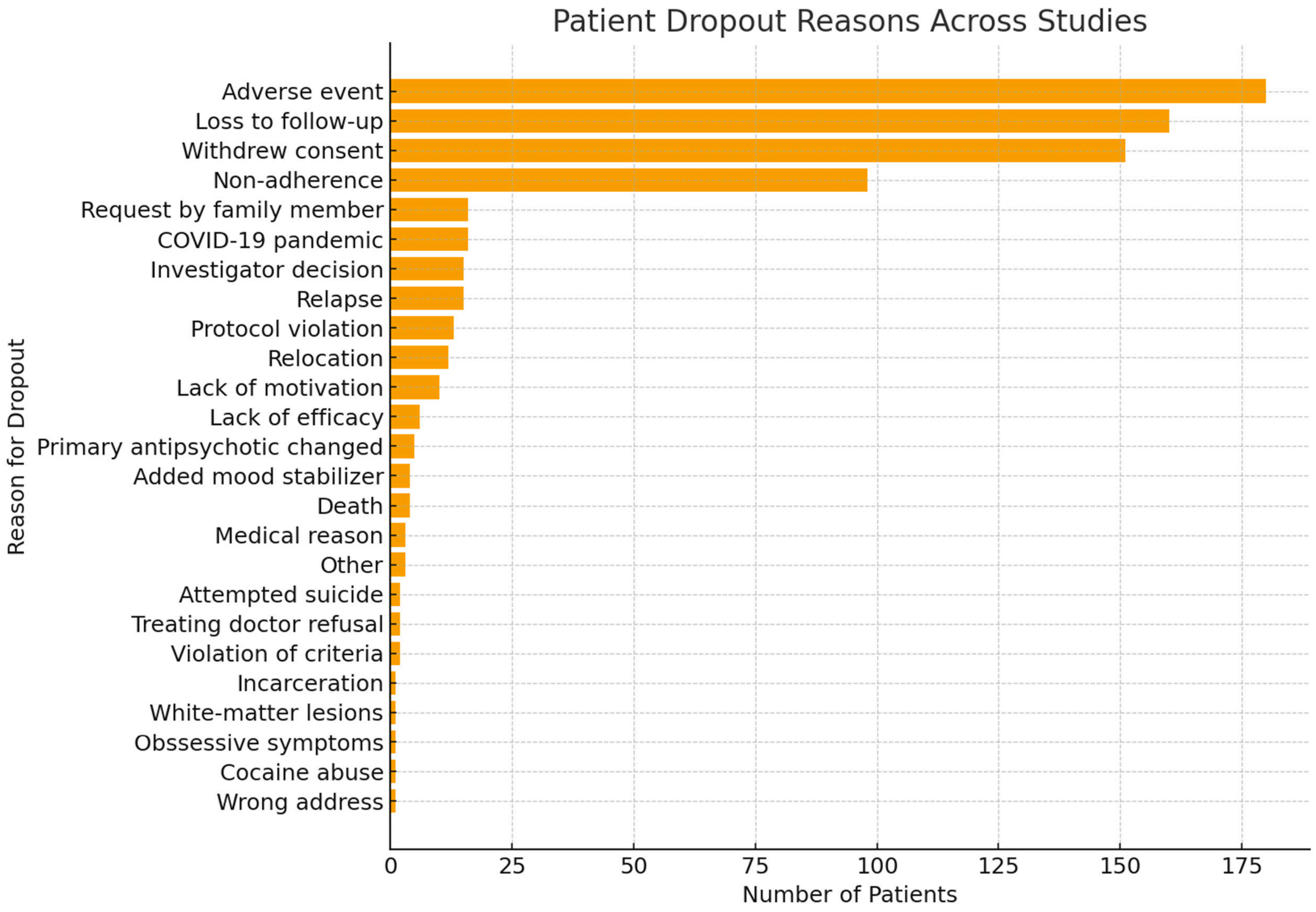

Supplementary Table S3. Detailed risk-of-bias via signaling questions for each individual study (Y = Yes, PY = Probably Yes, NI = No Information, PN = Probably No, N = No, NA = Not Applicable).

Study	Lenzi et al.2003	Geoff et al.2004	Friedman et al.2005	Sharma et al.2006	Zoccali et al.2007	Berk et al.2008	Levkovitz et al.2010	Lindenmayer et al.2010	Chaudhry et al.2012	Kane et al.2012	Usall et al.2014	Barnes et al.2016
Domain 1												
1.1 Was the allocation sequence random?	N	NI	NI	NI	NI	Y	NI	PY	Y	PY	Y	Y
1.2 Was the allocation sequence concealed?	N	NI	NI	NI	NI	Y	NI	PY	Y	PY	Y	Y
1.3 Did baseline differences between intevention groups suggest a randomization problem?	NI	N	NI	N	N	N	NI	N	N	N	N	N
Risk-of-bias judgement	High	Some concerns	Some concerns	Some concerns	Some concerns	Low	Some concerns	Low	Low	Low	Low	Low
Domain 2												
2.1 Were participants aware of their assigned intervention during the trial?	Y	N	NI	N	N	N	N	N	N	PN	N	N
2.2 Were carers and people delivering the	Y	N	NI	N	N	N	N	N	N	PN	N	N

interventions aware of the paricipants' assigned intervention during the trial?												
If Y/PY/NI to 2.1 or 2.2<br>2.3 Were the deviations from intented interventions due to trial context?	N	NA	PN	NA	NA	NA	NA	NA	NA	NA	NA	NA
If Y/PY to 2.3<br>2.4 Were these deviations likely to have affected the outcome?	NA	NA	NA	NA	NA	NA	NA	NA	NA	NA	NA	NA
If Y/PY to 2.4<br>2.5 Were these deviations balanced between groups?	NA	NA	NA	NA	NA	NA	NA	NA	NA	NA	NA	NA
2.6 Was an appropriate analysis used to estimate the effect of assignment to intervention?	N	NI	NI	NI	N	Y	Y	Y	Y	N	Y	Y
If Y/PY/NI to 2.6<br>2.7 Was there	Y	N	PN	PN	PN	N	N	N	N	PN	N	N

potential for a substantial impact (on results) of the failure to analyse participants in the group to which they were randomized?												
Risk-of-bias judgement	High	Some concerns	Some concerns	Some concerns	Some concerns	Low	Low	Low	Low	Some concerns	Low	Low
Domain 3												
3.1 Were data for this outcome available for all,<br>or nearly all participants who were randomized?	N	N	Y	Y	N	N	N	N	N	N	N	N
If N/PN/NI to 3.1<br>3.2 Is there evidence that the result was not biased by missing outcome data?	N	PN	NA	NA	N	N	N	N	Y	N	N	N
If N/PN to 3.2<br>3.3 Could missingness in the outcome depend on its true value?	PY	PY	NA	NA	PY	PY	PY	PY	NA	PY	PY	PY

If N/PN to 3.3<br>3.4 Is it likely that missingness in the outcome depended on its true value?	PY	PN	NA	NA	PN	PN	PY	PN	NA	PN	PN	PY
Risk-of-bias judgement	High	Some concerns	Low	Low	Some concerns	Some concerns	High	Some concerns	Low	Some concerns	Some concerns	High
Domain 4												
4.1 Was the method of measuring the outcome inappropriate?	PN	N	N	N	N	N	N	N	N	N	N	N
4.2 Could measurement or ascertainment of the outcome have differed between intervention groups?	N	N	N	N	N	N	N	N	N	N	N	N
If N/PN/NI to 4.1 and 4.2<br>4.3 Were outcome assessors aware of the intervention received by study participants?	Y	N	NI	N	N	N	N	N	N	PN	N	N
If Y/PY/NI to 4.3<br>4.4 Could assessment	PY	NA	PY	NA	NA	NA	NA	NA	NA	NA	NA	NA

of the outcome have been influenced by knowledge of the intervention received?												
If Y/PY/NI to 4.4 4.5 Is it likely that assessment of the outcome was influenced by knowledge of the intervention received?	PN	NA	PN	NA	NA	NA	NA	NA	NA	NA	NA	NA
Risk-of-bias judgement	Some concerns	Low	Some concerns	Low	Low	Low	Low	Low	Low	Low	Low	Low
Domain 5												
5.1 Were the data that produced this result analysed in accordance with a pre-specified analysis plan that was finalized before unblinded outcome data were available for analysis?	N	Y	PN	Y	Y	Y	Y	Y	Y	Y	Y	Y
5.2 Is the numerical result being assessed likely to have been selected,	Y	NI	PN	N	N	N	N	N	PN	N	N	N

on the basis of results, from multiple eligible outcome measurements within the outcome domain?												
5.3 Is the numerical result being assessed likely to have been selected, on the basis of results, from multiple eligible analyses of the data?	N	N	N	N	N	N	N	N	PN	N	N	N
Risk-of-bias judgement	High	Some concerns	Some concerns	Low	Low	Low	Low	Low	Low	Low	Low	Low

Supplementary Table S3 (Continued).

Study	Hirayasu et al.2016	Pawełczyk et al. 2016	Veerman et al.2016	Kumar et al.2017	Conus et al.2018	Deakin et al.2018	Goff et al.2019	Neill et al.2022	Shi et al.2022	Zhou et al.2022	Lang et al.2023	Anand et al.2024
Domain 1												
1.1 Was the allocation sequence random?	PN	Y	Y	PY	Y	Y	Y	Y	Y	Y	Y	PN
1.2 Was the allocation sequence concealed?	PN	Y	Y	PY	Y	Y	Y	Y	PY	PY	PY	PN

1.3 Did baseline differences between intevention groups suggest a randomization problem?	NI	N	Y	N	N	N	N	N	PN	N	N	NI
Risk-of-bias judgement	High	Low	Low	Low	Low	Low	Low	Low	Low	Low	Low	High
Domain 2												
2.1 Were participants aware of their assigned intervention during the trial?	Y	N	N	N	N	N	N	N	N	N	Y	Y
2.2 Were carers and people delivering the interventions aware of the paricipants' assigned intervention during the trial?	Y	N	N	N	N	N	N	N	N	N	N	Y
If Y/PY/NI to 2.1 or 2.2 2.3 Were the deviations from intented interventions due to trial context?	NI	NA	NA	NA	NA	NA	NA	NA	NA	NA	NI	PY
If Y/PY to 2.3 2.4 Were these	NA	NA	NA	NA	NA	NA	NA	NA	NA	NA	NA	PY

deviations likely to have affected the outcome?												
If Y/PY to 2.4<br>2.5 Were these deviations balanced between groups?	NA	NA	NA	NA	NA	NA	NA	NA	NA	NA	NA	PN
2.6 Was an appropriate analysis used to estimate<br>the effect of assignment to intervention?	N	Y	Y	Y	Y	Y	Y	Y	Y	Y	Y	Y
If Y/PY/NI to 2.6<br>2.7 Was there potential for a substantial impact (on results) of the failure to analyse participants in the group to which they were randomized?	NI	N	N	N	N	N	N	N	N	N	N	N
Risk-of-bias judgement	High	Low	Low	Low	Low	Low	Low	Low	Low	Low	Some concerns	High
Domain 3												
3.1 Were data for this outcome available for all,<br>or nearly all participants who were randomized?	N	Y	N	N	N	N	N	N	N	N	N	N

If N/PN/NI to 3.1 3.2 Is there evidence that the result was not biased by missing outcome data?	N	NA	N	N	PN	N	PN	N	N	N	N	N
If N/PN to 3.2 3.3 Could missingness in the outcome depend on its true value?	PY	NA	PY	PY	PY	PY	PY	PY	PY	PY	Y	PY
If N/PN to 3.3 3.4 Is it likely that missingness in the outcome depended on its true value?	PN	NA	PN	PN	PN	PN	PN	PN	PN	PN	PY	PN
Risk-of-bias judgement	Some concerns	Low	Some concerns	Some concerns	Some concerns	Some concerns	Some concerns	Some concerns	Some concerns	Some concerns	High	Some concerns
Domain 4												
4.1 Was the method of measuring the outcome inappropriate?	N	N	N	N	N	N	N	N	N	N	N	N
4.2 Could measurement or ascertainment of the outcome have differed between intervention groups?	N	N	N	N	N	N	N	N	N	N	N	N
If N/PN/NI to 4.1 and 4.2	Y	N	N	N	N	N	N	N	N	N	N	Y

4.3 Were outcome assessors aware of the intervention received by study participants?												
If Y/PY/NI to 4.3<br>4.4 Could assessment of the outcome have been influenced by knowledge of the intervention received?	PY	NA	NA	NA	NA	NA	NA	NA	NA	NA	NA	PN
If Y/PY/NI to 4.4<br>4.5 Is it likely that assessment of the outcome was influenced by knowledge of the intervention received?	NI	NA	NA	NA	NA	NA	NA	NA	NA	NA	NA	NA
Risk-of-bias judgement	High	Low	Low	Low	Low	Low	Low	Low	Low	Low	Low	Low
Domain 5												
5.1 Were the data that produced this result analysed in accordance with a pre-specified analysis plan that was finalized before unblinded outcome	Y	Y	Y	N	Y	Y	Y	Y	Y	Y	Y	PY

data were available for analysis?												
5.2 Is the numerical result being assessed likely to have been selected, on the basis of results, from multiple eligible outcome measurements within the outcome domain?	N	N	N	PY	N	N	N	N	N	N	N	N
5.3 Is the numerical result being assessed likely to have been selected, on the basis of results, from multiple eligible analyses of the data?	N	N	N	PY	N	N	N	N	N	N	N	N
Risk-of-bias judgement	Low	Low	Low	High	Low	Low	Low	Low	Low	Low	Low	Low

Important note: In the context of this review, we focus on efficacy and relapse prevention rather than other outcomes, such as safety and tolerability. In certain studies, efficacy outcomes are defined as secondary, albeit present in the publications. The RoB2 tool is outcome specific. Our risk-of-bias assessment of these studies concerns ONLY the efficacy-related outcomes.

Moreover, in certain studies, patients were randomized with respect to dosage for the same drug, which is more relevant when comparing safety and tolerability between different dosages. For comparing efficacy, randomization into a treatment and a placebo group is more suitable. We chose to mark such studies with PN in our assessment of randomization related bias.
